# Enabling FAIR data stewardship in complex international multi-site studies: Data Operations for the Accelerating Medicines Partnership® Schizophrenia Program

**DOI:** 10.1038/s41537-025-00560-x

**Published:** 2025-04-03

**Authors:** Tashrif Billah, Kang Ik K. Cho, Owen Borders, Yoonho Chung, Michaela Ennis, Grace R. Jacobs, Einat Liebenthal, Daniel H. Mathalon, Dheshan Mohandass, Spero C. Nicholas, Ofer Pasternak, Nora Penzel, Habiballah Rahimi Eichi, Phillip Wolff, Alan Anticevic, Kristen Laulette, Angela R. Nunez, Zailyn Tamayo, Kate Buccilli, Beau-Luke Colton, Dominic B. Dwyer, Larry Hendricks, Hok Pan Yuen, Jessica Spark, Sophie Tod, Holly Carrington, Justine T. Chen, Michael J. Coleman, Cheryl M. Corcoran, Anastasia Haidar, Omar John, Sinead Kelly, Patricia J. Marcy, Priya Matneja, Alessia McGowan, Susan E. Ray, Simone Veale, Inge Winter-Van Rossum, Jean Addington, Kelly A. Allott, Monica E. Calkins, Scott R. Clark, Ruben C. Gur, Michael P. Harms, Diana O. Perkins, Kosha Ruparel, William S. Stone, John Torous, Alison R. Yung, Eirini Zoupou, Paolo Fusar-Poli, Vijay A. Mittal, Jai L. Shah, Daniel H. Wolf, Guillermo Cecchi, Tina Kapur, Marek Kubicki, Kathryn Eve Lewandowski, Carrie E. Bearden, Patrick D. McGorry, René S. Kahn, John M. Kane, Barnaby Nelson, Scott W. Woods, Martha E. Shenton, the Accelerating Medicines Partnership® Schizophrenia (AMP® SCZ), Tashrif Billah, Kang Ik K. Cho, Owen Borders, Yoonho Chung, Michaela Ennis, Grace R. Jacobs, Einat Liebenthal, Daniel H. Mathalon, Dheshan Mohandass, Spero C. Nicholas, Ofer Pasternak, Nora Penzel, Habiballah Rahimi Eichi, Phillip Wolff, Alan Anticevic, Kristen Laulette, Angela R. Nunez, Zailyn Tamayo, Kate Buccilli, Beau-Luke Colton, Dominic B. Dwyer, Larry Hendricks, Hok Pan Yuen, Jessica Spark, Sophie Tod, Holly Carrington, Justine T. Chen, Michael J. Coleman, Cheryl M. Corcoran, Anastasia Haidar, Omar John, Sinead Kelly, Patricia J. Marcy, Priya Matneja, Alessia McGowan, Susan E. Ray, Simone Veale, Inge Winter-Van Rossum, Jean Addington, Kelly A. Allott, Monica E. Calkins, Scott R. Clark, Ruben C. Gur, Michael P. Harms, Diana O. Perkins, Kosha Ruparel, William S. Stone, John Torous, Alison R. Yung, Eirini Zoupou, Paolo Fusar-Poli, Vijay A. Mittal, Jai L. Shah, Daniel H. Wolf, Guillermo Cecchi, Tina Kapur, Marek Kubicki, Kathryn Eve Lewandowski, Carrie E. Bearden, Patrick D. McGorry, René S. Kahn, John M. Kane, Barnaby Nelson, Scott W. Woods, Martha E. Shenton, Justin T. Baker, Sylvain Bouix, Justin T. Baker, Sylvain Bouix

**Affiliations:** 1 Department of Psychiatry, Brigham and Women’s Hospital and Harvard Medical School, Boston, MA USA; 2 Department of Psychiatry, Massachusetts General Hospital and Harvard Medical School, Boston, MA USA; 3 Institute for Technology in Psychiatry, McLean Hospital, Belmont, MA USA; 4 Department of Psychiatry, Harvard Medical School, Boston, MA USA; 5 Harvard Medical School, Boston, MA USA; 6 Department of Psychiatry and Behavioral Sciences and Weill Institute for Neurosciences, University of California San Francisco, San Francisco, CA USA; 7 Mental Health Service 116D, Veterans Affairs San Francisco Health Care System, San Francisco, CA USA; 8 Department of Psychology, Emory University, Atlanta, GA USA; 9 Department of Psychiatry, Yale University School of Medicine, New Haven, CT USA; 10 Connecticut Mental Health Center, New Haven, CT USA; 11 Department of Psychology, University of California Los Angeles, Los Angeles, CA USA; 12 Orygen, Parkville, VIC Australia; 13 Centre for Youth Mental Health, The University of Melbourne, Parkville, VIC Australia; 14 Department of Psychiatry, Icahn School of Medicine at Mount Sinai, New York, NY USA; 15 Northwell Health, Glen Oaks, NY USA; 16 Department of Psychiatry, Hotchkiss Brain Institute, University of Calgary, Calgary, AB Canada; 17 Department of Psychiatry, Perelman School of Medicine, University of Pennsylvania, Philadelphia, PA USA; 18 The Discipline of Psychiatry, University of Adelaide, Adelaide, SA Australia; 19 Basil Hetzel Institute, Woodville, SA Australia; 20 Washington University School of Medicine, St. Louis, MO USA; 21 Department of Psychiatry, University of North Carolina at Chapel Hill, Chapel Hill, NC USA; 22 Department of Psychiatry, Beth Israel Deaconess Medical Center and Harvard Medical School, Boston, MA USA; 23 Institute of Mental and Physical Health and Clinical Translation (IMPACT), Deakin University, Geelong, VIC Australia; 24 School of Health Sciences, University of Manchester, Manchester, UK; 25 Department of Psychosis Studies, King’s College, London, UK; 26 Department of Brain and Behavioral Sciences, University of Pavia, Pavia, Italy; 27 Department of Psychology, Northwestern University, Evanston, IL USA; 28 Douglas Research Centre, Montreal, QC Canada; 29 Department of Psychiatry, McGill University, Montreal, QC Canada; 30 IBM T.J. Watson Research Center, Yorktown Heights, NY USA; 31 Psychotic Disorders Division, McLean Hospital, Belmont, MA USA; 32 Department of Psychiatry, Donald and Barbara Zucker School of Medicine at Hofstra/Northwell, Hempstead, NY USA; 33 Institute of Behavioral Science, Feinstein Institutes for Medical Research, Northwell Health, Manhasset, NY USA; 34 Department of Radiology, Brigham and Women’s Hospital, Boston, MA USA; 35 Department of Software Engineering and Information Technology, École de technologie supérieure, Montreal, QC Canada

**Keywords:** Schizophrenia, Biomarkers

## Abstract

Modern research management, particularly for publicly funded studies, assumes a data governance model in which grantees are considered stewards rather than owners of important data sets. Thus, there is an expectation that collected data are shared as widely as possible with the general research community. This presents problems in complex studies that involve sensitive health information. The latter requires balancing participant privacy with the needs of the research community. Here, we report on the data operation ecosystem crafted for the Accelerating Medicines Partnership® Schizophrenia project, an international observational study of young individuals at clinical high risk for developing a psychotic disorder. We review data capture systems, data dictionaries, organization principles, data flow, security, quality control protocols, data visualization, monitoring, and dissemination through the NIMH Data Archive platform. We focus on the interconnectedness of these steps, where our goal is to design a seamless data flow and an alignment with the FAIR (Findability, Accessibility, Interoperability, and Reusability) principles while balancing local regulatory and ethical considerations. This process-oriented approach leverages automated pipelines for data flow to enhance data quality, speed, and collaboration, underscoring the project’s contribution to advancing research practices involving multisite studies of sensitive mental health conditions. An important feature is the data’s close-to-real-time quality assessment (QA) and quality control (QC). The focus on close-to-real-time QA/QC makes it possible for a subject to redo a testing session, as well as facilitate course corrections to prevent repeating errors in future data acquisition. Watch Dr. Sylvain Bouix discuss his work and this article: https://vimeo.com/1025555648.

## Introduction

### Overview of the Accelerating Medicines Partnership Schizophrenia (AMP SCZ) observational project

Extensive research has been dedicated to characterizing the clinical high-risk (CHR) syndrome and identifying predictors of the onset of a psychotic episode. Heterogeneity across symptoms and outcomes is significant, and estimating the individual level of risk for transition and other poor outcomes remains a challenge, partially due to the need for large datasets to enable precision medicine methods^1–3^. The overarching goal of the AMP SCZ initiative is to improve current approaches to define early stages of risk and predict the likelihood of progression to psychosis and other psychiatric disorders, and ultimately to fast-track the development of effective, early-stage treatments for people who are at risk for psychosis (see also Addington et al. paper in this Special Issue)^4^. To stratify this complex population, the AMP SCZ program aims to follow 2617 participants for a period of two years, who are between the ages 12-30, and from 43 study sites around the world. Each participant provides oral and written informed consent. The project was approved by the governing institutional review board at each site and is registered on clinicaltrials.gov (NCT05905003). Individuals are assessed across a wide range of domains, including clinical measures, electroencephalography (EEG)^5^, magnetic resonance imaging (MRI)^6^, neurocognition^7^, fluid biomarkers^8^, digital health technology^9^, and audiovisual (A/V) recordings^10^. In addition to these primary scientific goals, the AMP SCZ program is committed to open science practices and will share de-identified subject-level data acquired through this project with the research community. Coordinating data capture, processing, quality assurance, quality control (QA/QC), and dissemination for this complex project involving over 150 scientists across five continents is challenging. To address such complexities, we present our approach to implementing a flexible and efficient data management infrastructure to ensure that the data are of the highest quality for the AMP SCZ program and the community at large.

### DataOps and FAIR principles

The explosion of big data projects in science and business has challenged traditional data management practices and inspired the creation of a new and independent approach called DataOps, which aims to optimize the entire data lifecycle and recognizes the interconnectedness of all teams involved in data acquisition, stewardship, and analysis^11^. DataOps is defined as “*a set of practices, processes, and technologies that combines an integrated and process-oriented perspective on data with automation and methods from agile software engineering to improve quality, speed, and collaboration and promote a culture of continuous improvement”*^12^.

The three fundamental principles underpinning DataOps are:

- *Smooth, fast, and secure data flow* from the sites to a final central repository for dissemination: How quickly can we deliver high-quality data to stakeholders?
- *Rapid feedback loops* on data quality or data flow issues to all necessary parties (domain experts, project manager, and leadership, and sites).
- *Continuous improvement* of processes, from the refinement of data acquisition procedures, all the way to providing better tools for data analytics.

The emphasis on a smooth and rapid flow of data and feedback on QA/QC issues is critical to AMP SCZ’s ability to ensure the highest possible data quality. Rapid feedback provides the opportunity to refine standard operating procedures where needed and, as noted previously, to redo testing with patients when data fails QA/QC. This large-scale approach is, to our knowledge, unprecedented.

Another core mission of the AMP SCZ program is to share all data acquired to the research community via the NIMH Data Archive (NDA)^13^. To maximize the impact of this open science effort, we are following the ‘FAIR Guiding Principles for scientific data management and stewardship’ to improve the Findability, Accessibility, Interoperability, and Reuse of data, which has guided (open and controlled) data sharing initiatives to make data easier to find and use^14^. *Findability* ensures researchers can swiftly locate and identify relevant datasets amidst a vast sea of information. *Accessibility* refers to making data available to diverse users regardless of their geographical location or technical expertize. *Interoperability* involves facilitating seamless data integration across platforms, disciplines, and tools. Lastly, *Reusability* focuses on the long-term sustainability of research efforts, advocating for structured, well-documented data accompanied by clear metadata. These principles have been essential to successful open science initiatives, including flagship efforts funded by the National Institute of Mental Health (NIMH), such as the Adolescent Brain Cognitive Development (ABCD) study and the Human Connectome Projects (HCP)^15^. Through these initiatives, a qualified researcher can now access thousands of datasets from studies of the brain in health and disease. The AMP SCZ program contributes to this effort by embracing and implementing the FAIR principles in research practice to elevate data-driven scientific exploration.

Implementing DataOps and FAIR practices is no small task for the AMP SCZ program. The project involves one data processing analysis and coordination center (DPACC), 43 international sites managed by two research networks (ProNET at Yale overseeing 28 sites and PRESCIENT at Orygen, Australia coordinating 15 sites), each maintaining separate data capture infrastructures, as well as NIMH teams at the NIMH Data Archives (NDA) and the NIMH Repository & Genomics Resource (NRGR). Of note, both research networks worked closely with participating non-US sites to allow data transfer to the NDA, making all data acquired available to the wider research community. The dataset is also of unprecedented volume and variety of data types for prospective studies in psychiatry. Human subject data also requires additional safeguards to protect participants’ privacy^16^. While the principle of Accessibility aims to promote open data sharing, the inclusion of sensitive attributes necessitates stringent ethical safeguards to protect participants’ privacy. Striking this balance involves the thoughtful design of data access protocols, encryption techniques, and controlled mechanisms for data sharing to ensure that data remains available to researchers while safeguarding the confidentiality of individuals.

### Our contribution

In this paper, we describe our approach to Data Operations, including (1) project management and data management systems needed to coordinate and harmonize data capture across a group of over 150 scientists involved in this project, (2) selection of appropriate electronic data capture systems, (3) use of a unique data flow infrastructure that automatically aggregates, de-identifies, and performs QC and data processing in close-to-real-time, and (4) dissemination of the data to the members of the AMP SCZ consortium and the wider community via the NIMH Data Archive. We also review how Data Operations can support monitoring activities, such as identifying missing or inconsistent data and communicating this information with the sites and research networks.

The governance of the data flow and decision-making processes in larger-scale studies is also a hallmark achievement of the AMP-SCZ project, where robust frameworks are used to oversee the intricate data pipeline. Such governance structures ensure the integrity, security, and ethical handling of data across multiple sites and networks, multi-data types and time spans, and set a precedent for best practices in large-scale research initiatives.

## Methods

### Data coordination and monitoring

Quality data collection begins with the coordination and training of personnel and the standardization and harmonization of data collection forms and methodology. The AMP SCZ DPACC has overseen scheduling, recording, and note-taking for meetings, including each of the Data Domain Teams, which are co-led by a combination of network and DPACC research team members^17^. The data domains are listed on the AMP-SCZ website and include clinical assessments, neurocognition, neuroimaging, electrophysiology, fluid biomarkers, digital measures, and speech sampling^18^. Each Data Domain Team has a companion manuscript in this special issue. There is collaboration among the networks and DPACC on these teams and in collecting data for the overall consortium, which has been augmented by annual meetings—first virtual and then in-person—attended by faculty and research staff alike. The DPACC has also worked closely with NIMH and each of the Data Domain Teams in the iterative development of all standard operating procedures (SOPs), which include training and certification. These SOPs are the focus of each of the Data Domain Team manuscripts in this special issue. They are also available on the AMP SCZ website and described in the AMP SCZ “lived experience” manuscript by the Website and Outreach Workgroup (WOW) in this special issue^19^. The DPACC has also worked with the networks in the development and harmonization of the forms used to acquire data, with the highlight being the new Positive SYmptoms and Diagnostic Criteria for the Comprehensive Assessment of the At-Risk Mental States (CAARMS) Harmonized with the Structured Interview for Psychosis–Risk Syndromes (SIPS) CHR clinical assessment instrument (PSYCHS), which is key for case ascertainment, measures of symptom severity, and determining the primary outcome (i.e., psychosis transition)^20^. The PSYCHS assessment – and training materials for its administration – are available on the AMP SCZ website. The DPACC also works with the networks to oversee measurement fidelity and performance tracking (quality assurance/quality control), ensuring human subject protections and ethical practices (review of Institutional Review Board documentation, adverse event reporting, subject safety) and oversight and reporting of recruitment rates, demographics, and trends in data collection. These efforts have been critical to the Data Operations, enabling the harmonization of SOPs and data collection instruments (encompassing over 20,000 variables) across all 43 sites. Additionally, the tight coordination between teams, research networks, DPACC, and its Data Operations team has helped to establish QA/QC procedures that are continuously being improved, thereby increasing the quality of the acquired data.

### Data capture systems

The AMP SCZ program involves several assessment activities leading to multiple data types, including interviews, cognitive testing, EEG, brain imaging, blood samples, saliva samples, audio and video samples, and an optional digital component (actigraphy watch, and smartphone daily surveys and sensor data). All these data, except fluid samples, are gathered electronically and need appropriate electronic data capture systems. One emphasis of the project has been to collect data directly into these systems, thereby avoiding the collection of paper forms, which increases the velocity and quality of data flow and allows for easier remote monitoring activities. Given the heterogeneous nature of the raw data, an ecosystem of data capture tools is being used in AMP SCZ.

**Fig. 1:**
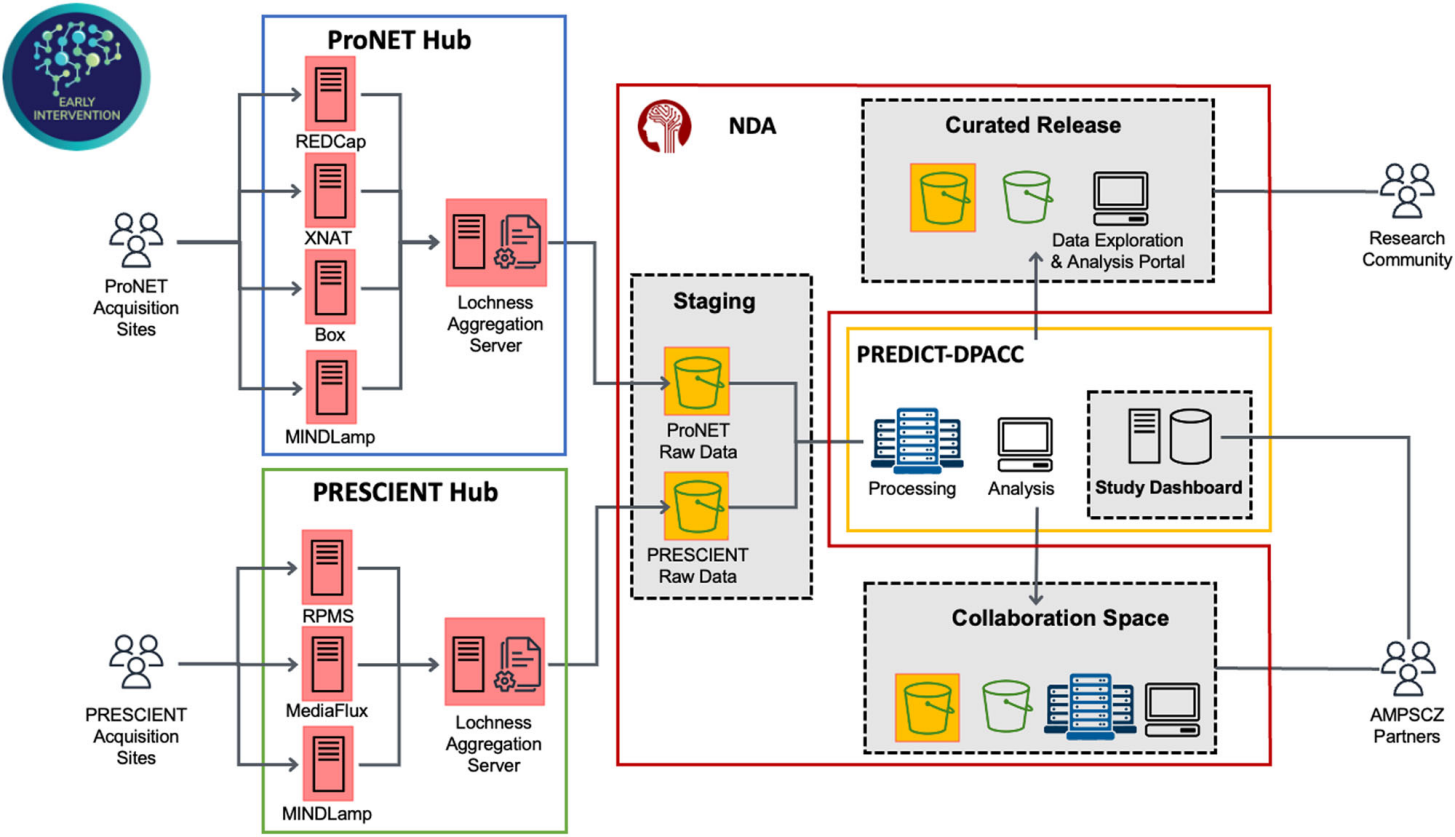
Diagram of the AMP SCZ data management system and flow. Data are captured, aggregated and de-identified within each research network before being transferred for processing and curation to the DPACC.

One unique aspect of AMP SCZ is that each network hub (ProNET at Yale and PRESCIENT at Orygen) is equipped with a different data capture ecosystem. The decision to maintain separate systems was rooted in a combination of factors, including sites and network hub familiarity with their respective systems, patient privacy protection, the time needed to put a new system in place, and financial constraints. An overview of the overall data management ecosystem is shown in Fig 1. We describe below the different systems being used in this project.

#### Web-based form entry

The primary database of this study consists of form data encompassing study management information (e.g., consent form documentation, inclusion criteria), clinical interviews (e.g., PSYCHS, NSI-PR), cognitive testing (e.g., Wechsler Abbreviated Scale Intelligence; WASI), and run sheets to document SOP adherence (e.g., MRI scan sheet, Blood collection run sheet, etc.)^20–22^.

ProNET uses REDCap (Research Electronic Data Capture), a secure, web-based application designed to facilitate data collection for research studies^23^. Developed by Vanderbilt University, it offers a versatile and user-friendly platform for researchers across various disciplines to create and manage databases, design surveys, and collect a wide array of data types efficiently. Its robust features include customizable data entry forms, advanced user access controls, audit trails for data tracking, and seamless data export capabilities for statistical analysis. REDCap ensures compliance with regulatory standards such as HIPAA, fostering data security and confidentiality.

PRESCIENT uses Orygen’s comprehensive Research Project Management System (RPMS), a proprietary, internally developed platform tailored to streamline every facet of study administration, encapsulating the entire research lifecycle –from participant recruitment, screening, scheduling, and assessments, to data capture—facilitated both directly by research participants and via research assistants, with an online system that offers a robust framework^24^. Rigorous audit trails meticulously document all data entries, ensuring transparency and accountability. Moreover, the RPMS includes a centralized documentation repository featuring approval and validation trails for study instruments and associated documentation, upholding meticulous standards in compliance with local and national privacy laws in Australia.

As these systems use fundamentally different internal structures, DPACC coordinated the creation of all forms. This process involved collaborating with domain experts from each team to build REDCap instruments that could be tested via DPACC’s internal REDCap system. One useful feature of REDCap is that individual instruments can be exported as CSV files and imported into a new system. We were, therefore, able to transfer the entire project directly into ProNET REDCap at Yale. For PRESCIENT, the RPMS team developed semi-automated procedures to convert the REDCap instruments into equivalent RPMS forms, which were then reviewed by the DPACC Data Operations team to check for any inconsistency between the codebooks. The current codebook has 24,940 fields that have all been harmonized between RPMS and REDCap.

#### MRI, EEG, actigraphy watch, and A/V recording data capture

For EEG, actigraphy watches data, and A/V recordings, there were no established database systems in either network. Given the tight timeline of this project, we decided to use cloud-based file hosting services for storing these data as they offer great flexibility in uploading and downloading data. PRESCIENT relies on MediaFlux, a secure and scalable platform for managing structured and unstructured digital assets, including sensitive research data^25^, while ProNET opted to use Box, a cloud-based content management system^26^.

Both MediaFlux and Box are, at their core, online folder systems that can be accessed from any device with an internet connection. Users can invite “collaborators” who can upload or modify files with extensive options for access controls. In the context of AMP SCZ, each site has a dedicated folder only accessible to them to deposit source data following the SOPs for a particular domain. PRESCIENT captures data with MediaFlux for MRI, EEG, actigraphy, and A/V recording data. ProNET uses Box for EEG, actigraphy, and A/V recording data, but uses a separate, dedicated system for MRI as outlined below.

#### MRI data capture

The system used for MRI data capture at ProNET is XNAT (Extensible Neuroimaging Archive Toolkit), an open-source imaging informatics platform developed by the Neuroinformatics Research Group at Washington University^27,28^. XNAT is a widely adopted platform in the neuroimaging community. It offers a robust infrastructure for the secure storage, organization, and analysis of diverse neuroimaging data types, such as MRI, positron emission tomography (PET), and computed tomography (CT) scans. PRESCIENT opted to store their neuroimaging data using MediaFlux, which serves the data storage requirements for this project but loses some of the advanced neuroimaging capabilities of XNAT (e.g., protocol validation and DICOM header de-identification).

#### The University of Pennsylvania Computerized Neurobehavioral Test Battery (PennCNB)

The PennCNB consists of a series of cognitive tasks that measure accuracy and speed of performance in major cognitive domains^29–32^. It is a web application that allows participants to perform tests from anywhere with an internet connection, eliminating the need for pencil-and-paper testing and manual scoring.

#### Smartphone data capture

Both ProNET and PRESCIENT use the MindLAMP platform, which can handle diverse smartphone-derived measures, including sensor data, user interactions, and app-generated information^33,34^. The platform is open source and can be deployed on various computing infrastructures. ProNET uses Amazon Web Services (the recommended cloud-based solution) to host MindLAMP, and PRESCIENT uses its own computing infrastructure.

### Data dictionaries and data organization principles

One complex challenge of this study has been the harmonization of all procedures, in particular, the need to have a single harmonized data dictionary for all measures across all sites. This was particularly complex, given the federated data capture ecosystem and two fundamentally different form data capture systems, REDCap and RPMS. We also had to build our codebook to facilitate sharing via the NDA, which has its own data dictionaries. We therefore used NDA conventions in our original codebook whenever a relevant NDA data structure existed. For forms without a matching NDA data structure, we followed the general NDA principles for naming variables. We later worked with the NDA to build their corresponding data structures (see Data Sharing section). One advantage of using the NDA data structures as a guideline for building a codebook is that they are freely available and used in other projects, thereby maximizing the chances of harmonization with projects beyond AMP SCZ. One should note this was a mostly manual process involving over 10 staff members carefully reviewing variable names and coding conventions to match between research networks (RNs) and also with the NDA.

**Fig. 2:**
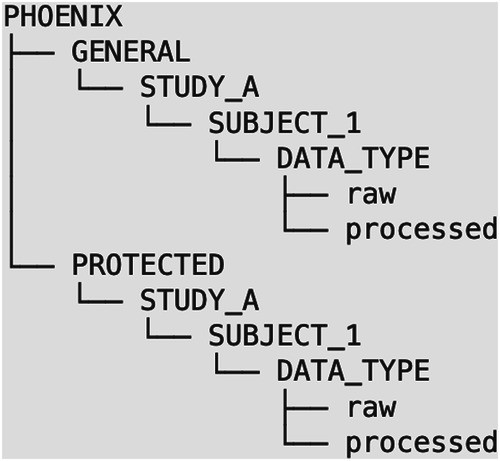
The PHOENIX directory structure categorizing multimodal data depending on whether they contain sensitive information.

Beyond the primary codebook for form data, we also store many associated files, such as MRI, EEG, A/V recordings, and more. To our knowledge, there is no established standard for storing the kind of multimodal data acquired in AMP SCZ. Existing standards such as the Digital Imaging and Communication in Medicine (DICOM) or Brain Imaging Data Structure (BIDS) tend to be domain-specific and do not handle well the heterogeneous nature of our data types and the heterogeneous frequency at which they are acquired. A close standard in spirit is the SPARC Data Structure, although it became public after the start of this project^35^. We therefore adapted a file organization convention developed by the Harvard Neuroinformatics Research Group, called the PHOENIX file system^36^. Data are stored in a data lake with folders organized hierarchically into sites, subjects, and data types. Additionally, at the root level of the PHOENIX file system, there are two subdirectories GENERAL and PROTECTED, thereby categorizing data depending on whether they contain sensitive information (see Fig. 2).

### Data flow and data security

#### Overview

As described above, the AMP SCZ data flow is unique because it aggregates data from two research networks, each with its own infrastructure as represented in Fig. 1. As described in detail in the Data Capture Systems, ProNET manages four data capture systems: REDCap, XNAT, MINDLamp, and Box, and PRESCIENT manages three systems: RPMS, MINDLamp, and MediaFlux. Both networks use the PennCNB system for neurocognition data. We developed aggregation and de-identification tools (see Lochness below) deployed on a server in each research network. Each server pulls data from the network’s respective data capture systems, organizes the data files on a local filesystem using the PHOENIX convention, and performs de-identification procedures. Aggregate and de-identified data are automatically transferred to a staging area at the NDA, from which the DPACC then pulls the data for review, validation, quality checking, and further processing. Data transfer is triggered every 8 h, and a monitoring dashboard is updated daily. The entire procedure is fully automated.

#### The Loch Ness aggregation system

Lochness is an open-source data management system designed by the Harvard Neuroinformatics Research Group to periodically aggregate data from various data archives into a local directory, also known as a data lake (hence the Lochness name). We extended the original system to support all data capture systems utilizing their Application Programming Interface (API). In the AMP SCZ project, Lochness is further configured to transfer selected data to a staging area (an S3 storage) maintained by the NDA and accessible by DPACC^37^. The software ensures robust encryption of credentials for connecting to each data capture system. Lochness arranges the collected data according to the AMP SCZ PHOENIX structure, categorizing data under “general” and “protected” data depending on whether these data contain sensitive information. This categorization is crucial for removing sensitive information from raw data, ensuring that they are not shared with the NDA. When transferring data from the local data lake to the NDA staging area, Lochness can be configured to selectively synchronize data types depending on agreements about the sharing of sensitive information.

#### De-identification procedures

A number of steps are taken to ensure sensitive information is handled adequately. For form data from RPMS and REDCap, sensitive data such as dates of birth are not exported from the source data capture systems. For other data types, specialized software has been built to de-identify data. We built a tool for anonymizing MRI header data, enabling teams to avoid inadvertently storing sensitive information such as patient and MR operator names, dates of birth, etc., thus significantly enhancing data privacy^38^. One complex workflow is the handling of A/V recordings. Per AMP SCZ policy, the identifiable raw recordings cannot leave the networks, and only de-identified QC features and redacted transcripts can be transferred to the NDA. We, therefore, developed pipelines to perform those tasks and installed them on the research networks aggregation server^39^.

#### Harmonizing form data

While data dictionaries are harmonized between REDCap and RPMS, the data representations differ. RPMS exports comma-separated values (CSV) files, each containing one instrument with data from multiple subjects, whereas REDCap exports a single JavaScript Object Notation (JSON) file per participant with all the instruments for that participant. To harmonize these data, daily exports from the RPMS database undergo a meticulous transformation to align all RPMS variables with their corresponding counterparts in the REDCap database. These harmonized data are then imported into a REDCap server maintained by DPACC, where the aggregate form data from both networks can be inspected. This rigorous matching procedure guarantees that both networks generate identical measures from their respective web-based forms, thereby ensuring data integrity and consistency across all sites.

Once data are transferred to the DPACC servers, they undergo validation, quality checking, and further processing (see Data Processing and QC). Summary information is then displayed on web-based dashboards for monitoring and visualization. Data are also uploaded regularly to the NDA collaboration space, where AMP SCZ partners can explore and analyze them in Amazon Workspaces virtual desktops. Data sharing with the community at large is done via traditional NDA data releases approximately every six months (see Data Dissemination, below).

#### Data processing and QC general principles and processes

The overarching goals of our QC processes are: close-to-real-time QC feedback, coverage of all domains, and an open science approach. All actionable QC findings are relayed to the appropriate teams (network hub management, domain expert, sites) via trackers and followed up for resolution by the DPACC and research networks monitoring and coordination teams. For fast QC feedback, we rely on automation whenever possible. For example, while a thorough visual inspection of an MRI may take days to complete with potential backlogs due to high demands on human resources, we run a full suite of automated tests that will raise alerts if data are missing or the scanning protocol is not followed (see below for details). This multi-layered QC approach, leveraging automation, allows for fast feedback and early detection of most errors. All data domains are covered by our QC procedures, and we are continuously improving our processes. Our first line of “QC coverage” often concentrates on missing data, followed by deeper QC measures such as cross-instrument consistency, accuracy of manual ratings on clinical measures, and much more. Some of these details are highlighted below. Finally, all our automated QC tools are open-source and available on the project AMP SCZ GitHub repositories for use by the general research community^40^.

##### Form QC

Form data include all interview and self-rated assessments and fluid data, as well as key information about the collection of MRI, EEG, cognition, and digital biomarkers. Once daily, all form data transferred to DPACC servers automatically enters a pipeline that generates outputs including QC measures that are uploaded to a custom study dashboard to visualize the quantity and quality of collected data. For example, different charts indicate the quantity of clinical data available at each site. In contrast, other charts indicate how many participants completed, did not complete, or skipped each PennCNB task. These visualizations are a cornerstone of real-time QC and monitoring, as they enable the identification of site-specific patterns related to potential challenges.

All form data are also automatically checked for missing data, with a focus on errors that can be immediately corrected or addressed because of this early detection. In addition to checking for missing values, numerous other checks are completed to ensure data collection is consistent and of the highest quality possible. For example, these include format checks (e.g., dates, barcodes); logic checks (e.g., dates are not in the future); outlier checks (e.g., IQ is not impossibly low); and cross-form consistency checks (e.g., if a participant endorses a symptom in one form, they should have a similar endorsement for that symptom in another form). In total, ~4500 variables and checks are examined for each participant on a daily basis. All identified flags/errors are then recorded in the QC tracker (spreadsheets) that indicates the participant ID, site, visit, form, flags, and date when issues were identified. These trackers are automatically updated daily and shared with coordinators and sites from both networks. By identifying and rapidly communicating incorrectly missing data and errors, sites are likely to be able to correct and address issues. This process results in less missing data and better documentation of reasons for why data are missing. Further, by systemically tracking and reporting errors, larger problems, such as protocol deviations are more likely to be caught and addressed, which improves the quality of future data collected. The ongoing detailed QC of form data has also led to the identification and improvement of inconsistencies across the large number of forms collected as part of AMP SCZ (e.g., dates, required fields) and in the harmonization of forms between REDCap and RPMS.

##### EEG

Data transferred to DPACC are automatically processed by pipelines that generate quality control metrics and data visualizations (e.g., EEG/ERP waves and topographic maps). Results are then presented on a secure web-based dashboard, allowing sites to review their own data. This dashboard is used in two weekly video conference meetings, scheduled to ensure the inclusion of all sites, regardless of their geographic location. These data are further reviewed by expert raters and scored on a scale of 1(=poor) through 4 (=excellent).

##### MRI

When new MRI data are transferred to DPACC, an automated QC pipeline generates a report within approximately 20 min, comparing the MRI parameters to a pre-qualified dataset from the same site. This enables DPACC to promptly identify any deviations from the scanning protocol. Alongside automated checks, DPACC research assistants visually inspect the MRI data, assigning a QC score (1=worst to 4=best) to each scan for comprehensive quality control. Details about this QC process are provided in the “The MR Neuroimaging Protocol for the AMP SCZ Consortium” article in this special issue.

##### MINDLamp

Research coordinators at each site can access the MINDLamp dashboard, allowing them to monitor the data collection status of each participant enrolled at their site. The dashboard enables coordinators to track the timestamps of all past survey submissions and the most recent batches of passive sensor data transferred to the MINDLamp server (i.e., geolocation and accelerometer) in real-time. In addition, DPACC provides a weekly QC report for network coordinators, summarizing the volume of sensor data received. The report includes a weekly QC summary of sensor data quality for each study participant on a scale of “Excellent,” “Average,” “Poor,” or “None.” This protocol guides site coordinators to identify participants with missing data and, when needed, initiate troubleshooting to diagnose whether the issue stems from non-compliance or technical difficulties.

##### Watch actigraphy

Actigraphy data are acquired with the Axivity AX3 device, a triaxial accelerometer, which participants are instructed to wear on their wrists. The device’s sampling frequency is configured to 12.5 Hz, a setting that ensures the device’s memory and battery retain functionality for a minimum duration of 30 days before reformatting and recharging. Subsequent to a 30-day period, participants must return the device during their next clinical appointment, at which point data extraction is conducted. Actigraphy files (.CWA format) are transferred to DPACC within a 12 h timeframe, where an analytical pipeline processes the raw data on a daily basis. This algorithm can discern periods during which the wrist device was not worn, generating minute-by-minute activity scores, and ultimately estimating sleep epochs based on identifying the prolonged low-activity period within the day. In addition, hourly activity scores are generated and uploaded to a custom-built study dashboard (DPDash—see description in “Data Visualization and Monitoring” section). Additionally, we present a monthly summary delineating data availability—expressed as the count of days for which any data were recorded—and data quality, gauged by the volume of hours for which data were captured on the days in question. These summaries are available to sites for review, facilitating ongoing monitoring and quality control.

##### A/V recordings

Audio and visual data are collected from open-ended qualitative interviews and semi-structured PSYCHS interviews, primarily utilizing the Zoom platform for recording. Additional audio data are gathered from daily diary entries submitted via the MindLAMP application. After interviews are conducted, staff members at the sites manually transfer the recording files to specific cloud storage services. Before processing these files, interview session details are logged into REDCap or RPMS run sheets. These electronic records document any deviations from the standard protocol and the overall quality of the recordings. Files are automatically transferred from the cloud storage services to aggregation servers hosted by the ProNET and PRESCIENT research networks. Upon arrival, an initial QC assessment is conducted, evaluating key attributes such as interview duration (ranging from 0 to 180 min), sound levels (between 40 and 90 dB), the number of faces in the videos, and the proportion of frames showing two faces. Any anomalies or deviations from set thresholds are flagged on the DPDash dashboard, triggering alerts to the research team. To be transcribed, audio files must satisfy criteria including a minimum duration of 10 min and a baseline sound level of 40 dB. In the absence of significant issues, the system assigns each interview a study day based on the interview date relative to the consent date. This method ensures privacy protection while maintaining the traceability of the data source. Post-transcription, the texts are analyzed for the number of inaudible words and for the presence of personally identifiable information (PII) or protected health information (PHI), which are redacted by human editors. These QC indicators are visually displayed on the DPdash system through color coding, facilitating the identification of issues. Regular summaries of these metrics are disseminated among the project team, and weekly QC meetings are convened to discuss findings and strategize enhancements.

### Data visualization and monitoring

An additional function of DPACC Data Operations has been the development of web-based data visualization tools for QC and monitoring purposes. We have developed several different platforms: (i) DPDash, a public-facing dashboard updated daily with information about data availability and QC information from all data domains^41^, (ii) an internal study dashboard, providing overview information about the project, aimed at tracking enrollment across sites, participant demographics within and across sites, and data missingness, and (iii) an EEG visual QC dashboard^42^.

DPdash is a Deep/Digital Phenotyping Dashboard designed to manage and visualize multimodal data coming in continuously over extended periods of time in individuals. At its core, DPdash is a web-viewable dashboard that allows users to visualize continuously acquired dynamic data coming in from multiple data streams, including smartphone-based passive and active data, surveys, actigraphy, MRI, EEG, etc. The goal of DPdash is to provide a streamlined visualization of ongoing data collection so that an investigator can determine what data have been acquired, what data are missing, and basic data patterns that are indicative of proper data collection. DPdash is extensible and can read any data if it conforms to a standard DPdash format.

Our internal study dashboard, a single HTML document written with Highcharts, Chart.js, and AG Grid, a software library for charting, provides information about participants’ demographics (race, age, IQ, socioeconomic status, sex) across sites and across phenotype (CHR vs community control), tracks enrollment over time, and data completeness across sites and instruments^43–45^. This is used weekly by the research networks to review potential undesired trends, such as imbalances in subject demographics between CHR and community controls.

**Fig. 3:**
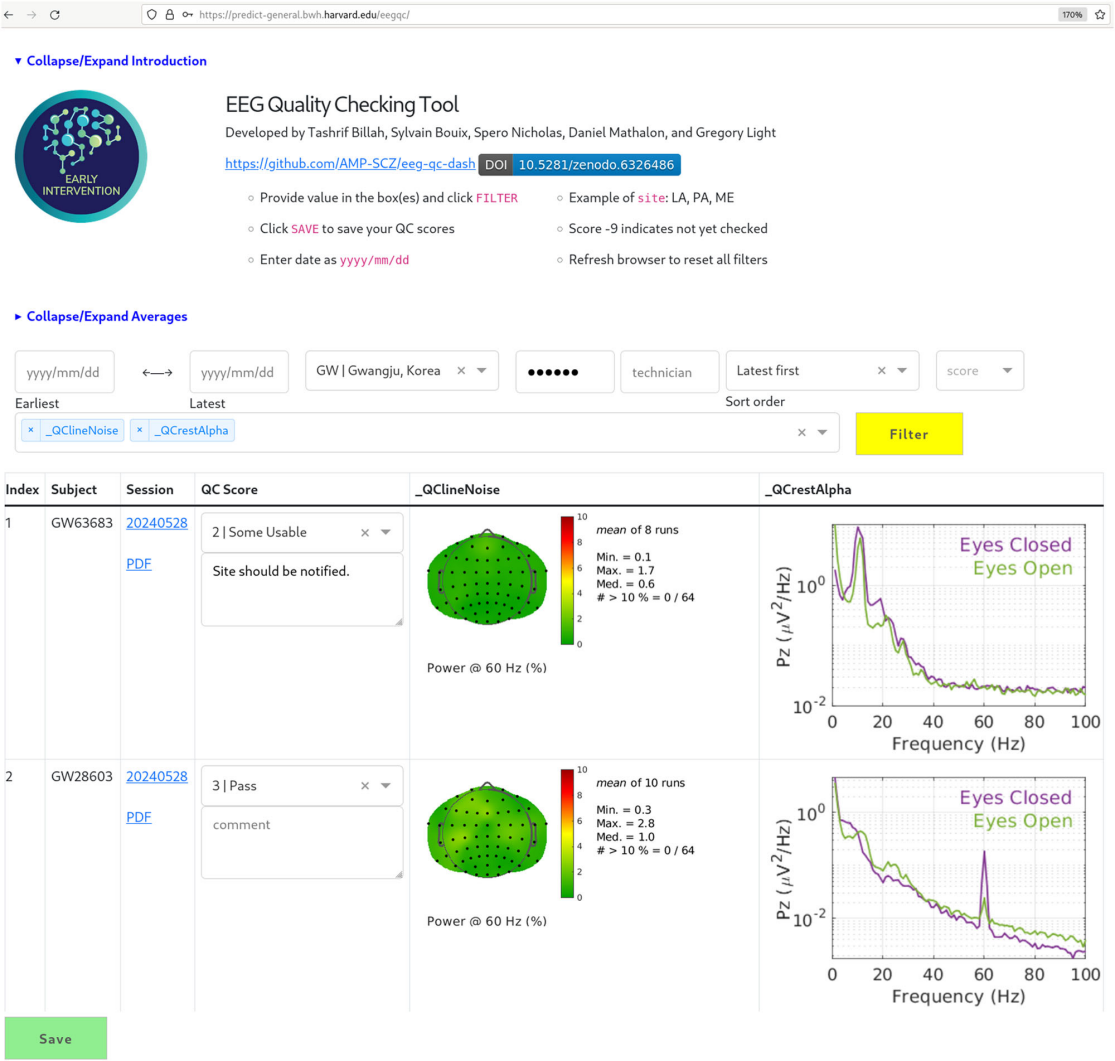
EEG quality checking tool. Raters are presented with a set of charts (e.g., line noise and response measures), which inform the QC rating captured in the application.

The *EEG Quality Checking Tool* is a web application written in Plotly/Dash^42^. It allows DPACC personnel to swiftly QC hundreds of EEG sessions. Each session is rated on a scale of 1(=poor) through 4(=excellent) based on various QC graphs. Common graphs include impedance and bridging among electrodes, noise in a power line, subject’s response time, task accuracy, and response measures (e.g., mismatch negativity). Reviewers can also insert comments justifying the score in a text box underneath. These features are illustrated in Fig. 3 below. The dashboard has other useful features such as filtering by date, site, technician, and score; sorting of the sessions alphabetically or chronologically; and viewing the run sheet of each session so that the rater has all information about the sessions in one place.

### Data dissemination via the NDA (collaboration space and public release)

As outlined in the Data flow section, the AMP SCZ data are being shared with consortium partners and the wider community. In collaboration with the NDA, we set up a special infrastructure for the AMP SCZ consortium partners to access recently acquired data. Any time we submit data to the NDA via their upload tool, they are deposited in a dedicated Amazon Web Services (AWS) s3 bucket. This bucket is then visible as a file system on virtual Linux desktops made available to AMP SCZ consortium members. The virtual desktops are created through AWS desktop virtualization service, Amazon Workspaces, and are managed by the NDA^46^. Workspace users can then inspect and analyze data directly through these virtual desktops without the need to download large amounts of data. DPACC provides example R and Python code to locate and inspect data, as well as specialized software for inspecting data types such as MRI.

In addition to these frequent uploads (target frequency is weekly) of recently acquired data, we curate a public data release (the target frequency is every 6 months). This curated release contains data carefully checked by domain experts available for download from the NDA web portal^47^.

## Results

Volume, velocity, and variety are integral dimensions that characterize the multifaceted nature of modern data landscapes. Volume refers to the sheer magnitude of data generated, collected, and processed, reflecting the exponential growth in information generation across diverse fields. The concept of velocity encapsulates the speed at which data are generated, shared, and updated, highlighting the dynamic nature of data flows and the need for close-to-real-time processing to extract timely insights. Variety underscores the diversity of the data types and formats, ranging from structured data in databases to unstructured content like text, images, and multimedia. These dimensions collectively underscore the challenges and opportunities presented by today’s data-rich environments, shaping strategies for data management, analysis, and utilization across disciplines and industries. Here, we report on the volume, velocity, and variety of data collected for the AMP SCZ initiative. We also report on human resources and time needed to put this infrastructure in place and maintain it while the project is ongoing.

### Volume

AMP SCZ is the largest prospective cohort study of CHR and boasts the largest data in this population. A single participant may have up to 11,324 variables collected in form data, two EEG sessions (mismatch negativity, auditory and visual P300, 40 Hz Auditory Steady State Response, and resting state), two MRI sessions each with anatomical, diffusion, and functional scans, two A/V recordings of open interviews each about 20 min long, as well as up to eight recordings of PSYCHS interviews. Additionally, this study continuously collects smartphone data for up to a year including geolocation, accelerometry, and screen state (on/off). Finally, we acquire precise accelerometry data through a wristband for up to a year. The combined amount of raw data for one participant completing all measures is about 50 GB (see Table 1 for details). As of February 2024, we have collected close to 15TB of raw data. Given the expected sample of approximately 2000 CHR individuals and 640 matched community controls, AMP SCZ will likely collect over 100TB of raw data. This does not account for the many derived data from our processing pipelines.

**Table 1:** The heterogeneous nature of the multimodal data acquired in AMP SCZ.

Domain	Frequency	Type	Format	Size
Form data (interviews, runsheet, cognition, etc)	monthly	tabular	JSON, CSV	10MB/participant
EEG	baseline & month 2	binary	BrainVision Core Data Format 1.0	1GB/session
MRI	baseline & month 2	binary	DICOM	5GB/session
Smartphone sensor	daily^a^	tabular	JSON	100MB/day collected
Actigraphy watch	monthly^a^	binary	CWA	200MB/month
A/V	Daily diaries (phone), Baseline & month 2 (open), Monthly (psychs)^b^	binary	WAV, MP3, M4A	200MB/h for video 20MB/hour for audio only 200MB/wav

^a^Smartphone and actigraphy watch data is sampled multiple times per second but transferred as a single data file daily (smartphone) and monthly (watch).

^b^Audio/video recordings of open interviews are collected twice (baseline and month 2), audio/video recordings of PSYCHS are collected up to 10 times (screening, baseline, month 1, 2, 3, 6, 12, 18, 24, and conversion), audio diaries can be recorded on smartphone at least daily.

### Velocity

**Fig. 4:**
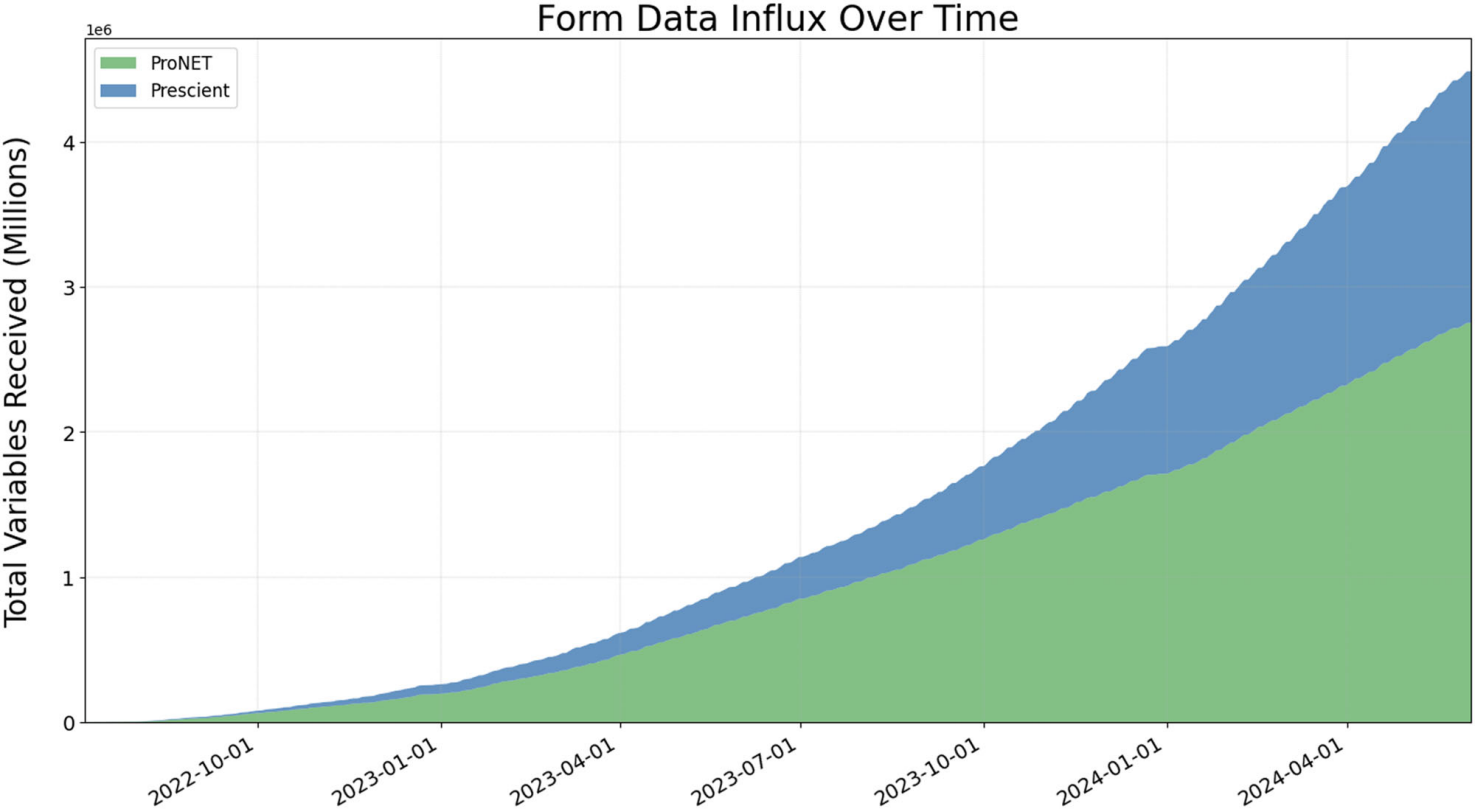
Total number of form data variables received over time.

One major focus of Data Operations has been to provide a rapid and reliable flow of information. We report here some outcomes related to the velocity of the various components of data flow and QC. First, data capture is done in real-time for all forms, except IQ measures that are administered with paper tests. Raw data files from MRI, EEG, A/V recording, Axivity device, are uploaded within 24 h of acquisition. Information about fluid biomarkers (vial storage information, CBC w/ differential) is captured within 24 h of sample collection. Data aggregation across both networks and transfer to DPACC infrastructure occurs every 6 h. As this prospective study is ongoing, the amount of data acquired weekly is increasing (see Fig. 4).

We run processing and QC pipelines every 24 h and feedback from automated QC workflows is provided to the research networks and sites within 48 h from data capture. This covers all data types (forms, MRI, EEG, A/V recordings, smartphone, Axivity). The DPDash study dashboard is updated daily as well.

EEG and MRI data undergo an additional visual inspection process, requiring more time to perform. For EEG, as of February 2024, there are 1480 sessions available, 1286 have been visually inspected (87%), 41 are under review (3%) and 153 need to be reviewed (10%). Among the 153 sessions waiting to be reviewed, 13 have been in the queue for 1–6 days, 14 for 7–13 days, 16 for 14–20 days, 9 for 21–27 days, and 101 for at least 28 days. For MRI, we have 1365 sessions available, 1219 have been visually inspected (90%), 146 are under review (9%). Among the 146, 16 have been in the queue for 1–6 days, 24 for 7–13 days, 18 for 14–20 days, 7 for 21–27 days, and 81 for at least 28 days. Reasons for the longer wait time (>28 days) for visual inspections are multiple, including missing data, partial data transfer, mismatched dates between the scan file and the run sheet, unexplained additional data not documented in the run sheet, and more.

This QC feedback has led to the correction of over 8,000 forms by the acquisition sites since the beginning of the project. The median time to resolve a flagged issue is 31 days, indicating that data are corrected before a participant starts their next visit.

### Variety of data types

This study features a comprehensive assessment battery and includes a wide range of data types. The diversity of data spans multiple domains, from the type of information collected, the frequency at which data is collected, the amount of data collected and the formats and standards used to store these datasets. Details are provided in Table 1.

### Dissemination

#### Data

The first curated data release containing screening and baseline data from 430 unique subjects occurred in November 2023. An updated dataset was released in June 2024, which comprises fully quality-controlled data from screening through month 2 for 1,048 unique subjects. This includes clinical and behavioral measures, with baseline data from 647 to 934 subjects, month 1 from 486 to 520 subjects, and month 2 from 529 to 552 subjects, depending on the measure. Unprocessed EEG data includes 640 subjects at baseline and 301 at month 2, and unprocessed magnetic resonance imaging (MRI, dMRI, and rs-fMRI) covers 425 subjects. Redacted open interview transcripts include transcripts of language samples collected at baseline for 532 subjects and month 2 for 275 subjects using the Open-ended language sample protocol and language samples collected using the PSYCHS clinical interview at screening for 132 subjects, baseline for 181 subjects, month 1 for 225 subjects, and month 2 for 214 subjects. Raw actigraphy watch data is available for 258 subjects, smartphone surveys for 174 subjects, and smartphone data (including screen use, accelerometry, and geolocation) for 110 subjects. Fluid biomarkers QC measures are available at baseline for 772 to 887 subjects and at month 2 for 445 to 507 subjects.

#### Resources requirements

The project funding started in September 2020, and the first participant started their baseline assessment in June 2022. A significant time-consuming part of this project was harmonizing the protocol across the two research networks. The data flow infrastructure took about one year to put in place and test. The core DataOps infrastructure team consists of two research engineers and one junior faculty. Additionally, domain-specific engineers/researchers were involved in establishing QC and processing pipelines. As a general rule, each domain requires one dedicated engineer and one scientific advisor. Once the project is ongoing, a minimum of two full-time engineers are needed to maintain and monitor data operations. Each data domain requires one dedicated technical staff to optimize processing pipelines and QC processes. Replicating this infrastructure for a different project would take 3–6 months before being operational, but if new pipelines are needed or a new protocol is put in place, the time to production would be 6–12 months depending on the complexity of the changes.

## Discussion

This complex project highlights the need for a flexible and agile infrastructure to enable the continuous flow and quality assurance of heterogeneous multimodal data. We have implemented an innovative framework capable of managing a federated data capture ecosystem from two research networks with sometimes fundamentally different data management designs. Data are harmonized following best practices from the NDA and disseminated to the community following an open science approach to maximize the impact of the largest prospective study in this population. One critical aspect of the data operations framework is the continuous flow of data and information both forward and backward across all partners. We aim to provide feedback and address data missingness and data quality concerns as quickly as possible to ensure no major issues are discovered months after data acquisition. This has necessitated the coordination and hard work of a community of over 150 scientists and research staff worldwide. We are continually learning and experimenting to improve our processes and workflows, including adding more telemetry of the entire data management infrastructure to ensure faster detection of issues, implementing and revising QC procedures to reduce manual checks and including more cross-modality information, as well as working with domain experts to improve processing pipelines.

While our infrastructure has shown to be invaluable for ensuring superior data quality and smooth data flow, it does have limitations and challenges. One important challenge was that ProNET, PRESCIENT and DPACC each sent a funding proposal with different designs and study protocols. As a result, the better part of the first year was spent on coordinating the design of a single harmonized protocol for the entire study. This meant, in some cases, changes in planned data capture systems (e.g., EEG hardware or smartphone data capture), schedule of assessments, and more. This involved coordinating 100+ scientists across the globe, and it took almost two years before we could test the first participant. Information systems also often fail in subtle ways, and monitoring all possible data transfer issues over three separate infrastructures is challenging and can lead to undetected partial data flow interruption. We also spent significant time designing custom visualization tools, even though existing tools such as Microsoft Power BI or Tableau would have likely met our needs with less programming overhead.

The immediate future steps of the data operations team for this project will be to curate derived data from several data types (e.g., brain volumes, language features, daily activity summaries, EEG peak amplitudes, etc.) that will be used by the data analysis group for the overarching goal of AMP SCZ to generate tools that will fast-track the development of effective, early-stage treatments for people who are at risk for schizophrenia.

## Supplementary information

AMP SCZ Member List & Affiliations

## Data Availability

The data used in this paper are available via scheduled releases at the NIMH Data Archive (NDA) AMP SCZ Data Repository (https://nda.nih.gov/ampscz).

## Code availability

All data flow tools are open source and available on GitHub (https://github.com/AMP-SCZ/ - currently 26 repositories). Many of the core applications, such as the data aggregation tool or visualization dashboard, could be used by others, although they would need to be better documented. We plan to continue developing these tools for the broader community. Some of the tools are specific to this project (e.g., some of the QC procedures are instrument-specific, and it is challenging to abstract them to be portable to other projects).
